# Gambling disorder and chronic diseases in Finland: a nationwide register study

**DOI:** 10.1093/eurpub/ckac129.554

**Published:** 2022-10-25

**Authors:** T Grönroos, AH Salonen, TA Latvala, A Kouvonen

**Affiliations:** 1 Department of Public Health and Welfare, The Finnish Institute for Health and Welfare, Helsinki, Finland; 2 Faculty of Social Sciences, University of Helsinki, Helsinki, Finland; 3 Faculty of Health Sciences, University of Eastern Finland, Kuopio, Finland; 4 Centre for Public Health, Queen’s University Belfast, Belfast, UK

## Abstract

**Background:**

Previous register-based studies on comorbidity have mainly focused on gambling disorder (GD) and psychiatric disorders. However, knowledge on somatic health of persons with GD is also needed. This nationwide register-based study aims to examine the gender-specific prevalence rates of chronic diseases and conditions among the Finnish adults with GD.

**Methods:**

This study utilizes aggregated data of persons aged 18 and over with GD diagnosis (corresponding to pathological gambling, ICD-10; F63.0) in 2011-2020. The data were retrieved from the Finnish nationwide health registers: Register of Primary Health Care visits and Care Register for Health Care, including specialised outpatient and inpatient health care, and inpatient social care. All diagnostic groups were included. Corresponding figures for the total population with same age range were presented as reference numbers.

**Results:**

The preliminary results showed that 2,617 persons with the median age of 33.5-36.0 were diagnosed with GD (men n = 1,858; women n = 759). Despite the fact that the prevalence rates of the general population were not age-adjusted, many chronic diseases and conditions were more prevalent among persons with GD compared with the general population. The prevalence rates of psychiatric disorders (87.5% vs 29.2%) and nervous system diseases (23.9% vs 15.2%) were particularly high. Musculoskeletal diseases (61.6% vs 55.8%) and digestive diseases (30.2% vs 27.6%) were also slightly more prevalent. Memory disorders (1.1% vs 5.3%), cardiovascular diseases (25.3% vs 41.0%), and cancer (15.6% vs 24.4%) were less prevalent. Among persons with GD, all comorbid diseases were more prevalent among women than among men.

**Conclusions:**

Psychiatric disorders and nervous system diseases are exceptionally prevalent in persons with GD. These findings highlight the need for health and social care professionals to recognize that persons with GD may additionally have other disorders that need attention.

**Key messages:**

• Mental disorders are the most common comorbidity among persons with GD, however, many somatic chronic diseases are also common.

• Comorbidities are more common among women with GD than among men with GD.

